# Evaluating Modular Approach to Therapy for Children with Anxiety, Depression, Trauma and Conduct Problems (MATCH-ADCT) in Norwegian child and adolescent outpatient clinics: Study protocol for a randomized controlled trial

**DOI:** 10.1186/s13063-018-3074-9

**Published:** 2019-01-07

**Authors:** Kristine Amlund Hagen, Asgeir Røyrhus Olseth, Hanne Laland, Kristian Rognstad, Anett Apeland, Elisabeth Askeland, Knut Taraldsen, Bernadette Christensen, John Kjøbli, Ana M. Ugueto, Sarah Kate Bearman, John Weisz

**Affiliations:** 1 The Norwegian Center for Child Behavioral Development (NCCBD), a University of Oslo affiliate, Postboks 7053, Majorstuen, 0306 Oslo, Norway; 2 Regional Center for Child and Adolescent mental health, Eastern and Southern Norway, Nydalen, Postboks 4623, 0405 Oslo, Norway; 3 Department of Psychiatry and Behavioral Sciences, The University of Texas Health Science Center at Houston, McGovern Medical School, 7000 Fannin St #1200, Houston, TX 77030 USA; 4 Department of Educational Psychology at the University of Texas at Austin, 1912 Speedway Stop D5800, Austin, TX 78712 USA; 5 Department of Psychology, Faculty of Arts and Sciences, Harvard University, 1030 William James Hall, 33 Kirkland Street, Cambridge, MA 02138 USA

**Keywords:** Effectiveness study, Depression, Anxiety, Conduct problems, Trauma, Children and adolescents, Evidence-based treatment, Modular approach, Implementation, Measurement feedback system

## Abstract

**Background:**

Norwegian health, care, and welfare services are experiencing increased demands to deliver services that are safe, effective, of high quality, and that ensure user involvement. Yet, evidence-based treatment for common disorders such as depression, anxiety, trauma, and behavioral problems in children are not regularly used in clinical practice in Norway. Possible explanations for this are that many standard, evidence-based treatments may have difficulty addressing the complexity and comorbidity of referred children and the fact that children’s treatment needs often shift during treatment. The Modular Approach to Therapy for children with Anxiety, Depression, Trauma and Conduct problems (MATCH-ADTC) was designed to address these challenges and reduce some of the barriers to therapists’ use of evidence-based treatment in their practice.

**Methods/design:**

Participants will include 280 children (aged 6–14.5 years at intake) who receive treatment in child and adolescent mental health outpatient clinics in Norway, and their families. Families are randomly assigned to either the experimental group receiving treatment from therapists trained in MATCH, or to the comparison group receiving treatment from therapists delivering treatment as usual (TAU). Data on children’s symptomology, child and family functioning, demographics, background information, and mental health outcomes are collected as well as frequent feedback on treatment response, plus video-recordings of treatment sessions and implementation quality scores from each participating clinic. Questionnaires are administered in six waves.

**Discussion:**

MATCH has been tested in the US with promising results, but we do not know whether this treatment approach will produce similar results in Norway. The implications of this study are

1. Possibly better treatment outcomes and/or more efficient improvements for children and families treated in mental health outpatient clinics in Norway
2. Clinicians learning to use more evidence-based practices in their treatment
3. Implementation of standard procedures for obtaining feedback from children and families and sharing the feedback with clinicians
4. Increased understanding, at the end of the trial, of whether introducing MATCH improves outcomes for children and families treated in mental health outpatient clinics

**Trial registration:**

ISRCTN, registration number: ISRCTN24029895. Registered on 8 August 2016.

**Electronic supplementary material:**

The online version of this article (10.1186/s13063-018-3074-9) contains supplementary material, which is available to authorized users.

## Background

The lack of congruence between what is being delivered in health, care, and welfare agencies, and the best available empirical knowledge, has long been a major challenge [17]. There is reason to believe that these agencies can deliver services of higher quality given better procedures for implementing research findings in practice. Moreover, the health, care, and welfare services in Norway are now experiencing greater demands to deliver services that are safe, effective, and of high quality, and that ensure user involvement. Steps toward meeting these requirements likely include the supply and translation of relevant empirical knowledge, new work methods, and the establishment of user-feedback systems. A premise in this regard is the ability and willingness of agencies to implement empirically supported practice and innovations, as is the implementation of new and promising treatments for vulnerable children and their families, evaluating the effects of these treatments, and monitoring user pathways.

According to Norwegian national statistics, depression, anxiety, conduct problems, and trauma-related difficulties combined make up 57% of the 10 most frequent referral reasons to specialized mental health outpatient clinics for children and youth aged 0–18 years [8]. The reasons for referral vary with the patients’ age and gender. Results from a survey commissioned by the Directorate of Health in 2008, which included 90% of all outpatient specialized mental health clinics in Norway, showed that 49% of boys referred to specialized care were in the 6–12 years age range compared to 26% of the girls. In adolescence, the picture was different: 56% of girls and 42% of boys referred to specialized outpatient clinics were between the ages of 13 and 17 years. Conduct problems are the most frequent reason for referral for boys, followed by hyperactivity/attention problems and depressive disorders. Depressive symptoms are the most frequent reason for referral for girls, followed by hyperactivity/attention problems, conduct problems, and anxiety [3]. Comorbidity, or the presence of symptoms of multiple disorders, is quite common in children and youth. In US adolescents (13–18 years of age), it has been found that about 40% of youth who suffer from one group of mental disorders also meet the criteria for another group of mental disorders [14]. There is reason to believe that similar rates of comorbidity are present in Norwegian samples; for example, in a population-based study of 8–10-year-olds in the city of Bergen, Norway, of all children with a psychiatric disorder, 26% had a comorbid condition [11]. Moreover, among children aged 6–12 years participating in a Norwegian foster home study, 63.4% of children with a psychiatric diagnosis met the criteria for additional disorders [13].

In general, evidence-based treatments (EBTs) for depression, anxiety, and behavioral problems in children are not regularly used in clinical practice [22]. One explanation for this may be that many standard evidence-based treatments are designed to focus on a single problem or disorder (or homogeneous cluster of similar problems), and may have difficulty addressing the comorbidity that is common among referred children as well as the tendency for patients’ needs to shift during treatment. In addition, EBTs often have a predetermined order of session contents, and this standardization may limit their flexibility in addressing comorbidity and fluctuations in treatment needs during episodes of care. Some evidence-based programs that have been developed and tested primarily with children recruited to research-based facilities may also face limitations when implemented and delivered in everyday clinical practice. Finally, most clinicians work with several patients simultaneously who often present very different symptoms, making it difficult for the clinicians to make good use of — or commit fully to – one single-syndrome intervention. MATCH, through its transdiagnostic, modular design, seeks to reduce some of the barriers to therapists’ use of empirically supported intervention in their practice [4].

### Description of MATCH-ADTC

The overall treatment approach, called Child STEPs, includes the MATCH treatment manual and a monitoring and feedback system, called Progress Assessment in Therapy (or PATH). The monitoring and feedback system includes a model for clinical consultation for reviewing clinical progress, planning for next session and preparing specific interventions skills. In the Norwegian MATCH trial an extra level of consultation has been added to facilitate possible language barriers and cultural adaptation to clinical practice. MATCH brings together procedures of EBT for anxiety, depression, trauma, and behavioral problems. In the original protocol, these procedures were grouped within 33 separate modules (intervention elements). In the Norwegian trial, two psychoeducation modules have been added to the treatment protocol, including one addressing “Learning about Traumatic Stress for Children” and one addressing “Learning about Traumatic Stress for Parents.” There were also some protocol adaptations made in order to better fit Norwegian cultural norms.

PATH uses frequent brief assessments to inform clinicians about each child’s treatment response, week by week, throughout treatment, thereby supporting clinicians’ efforts to adjust and personalize intervention throughout the episodes of care. Core features of the treatment program include:

- A menu of treatment modules, each describing one intervention element that is used frequently in EBTs
- MATCH modules grouped within the four problem areas: anxiety, depression, trauma, and conduct problems
- MATCH flowcharts to guide clinician decision-making throughout treatment
- Clinician decision-making informed through PATH, which includes brief weekly assessments of internalizing, externalizing, and ratings of top problems as reported by children and their caregivers
- Results of the weekly assessments summarized and posted on a web-based platform, forming a “clinical dashboard” for each child
- These dashboards are accessed by clinicians and consultants in weekly case consultation and used to gauge treatment response and plan adjustments in treatment

Therapists who are trained in MATCH are taught to combine modules based on the child’s needs and problems as they progress through treatment.

### Earlier findings

In an earlier randomized controlled trial from 2012 conducted in the US, MATCH was compared to two other conditions: treatment as usual (or “usual care”; TAU) and standard EBTs [23]. The participants were clinic-referred children, aged 7–13 years, who had been referred through normal community channels. The children were treated by clinical practitioners in the clinical service settings where those practitioners worked. Results of this trial indicated that children and families receiving MATCH scored more favorably and improved faster on several main outcome measures than did children and families in the two other treatment conditions. The measures included child- and parent-reported measures of internalizing, externalizing, and total problems, as well as child- and parent-report measures of the severity of the “top problems” identified by children and parents prior to treatment.. Effect sizes for child- and parent-report measures combined ranged from *d* = .48 to .70. Furthermore, at treatment termination, children receiving MATCH were diagnosed with fewer psychiatric conditions than were children who received usual care.

A second study [7] reported outcomes from the Weisz et al. [23] randomized trial over a 2-year follow-up period. The standardized child-report and parent-report measures used assessed internalizing, externalizing, and total child problems at 3-month intervals over the course of 2 years. The measures showed rates of improvement to be significantly superior for MATCH, relative to usual care, with effect sizes for child and parent measures combined ranging from .51 to .65. MATCH was also numerically superior to usual care on these measures (effect sizes ranging from .33 to .37), but those differences were not statistically significant.

A third study [6] was a randomized trial testing MATCH in comparison to “community implemented treatment” (CIT, which was a government-supported implementation of multiple evidence-based practices for children). The sample included clinic-referred children aged 5–15 years of age, treated by community clinical practitioners in the clinical service settings where those practitioners worked. In this trial, as in previous findings, children treated with MATCH showed significantly faster rates of improvement over time on the primary outcomes than did children in the CIT condition, with effect sizes ranging from .38 to .56. These outcomes included child- and parent-reported internalizing, and total problems, as well as severity ratings on the top problems identified by children and parents, respectively. Analyses also showed that children treated with MATCH, compared to the CIT children, required less time in treatment, were less likely to receive additional treatment services, and were less likely to use a variety of psychotropic medications during the treatment phase of the study.

### Aims

The aims of the current trial are:

- To evaluate the effectiveness of MATCH therapy on children and families referred to, and treated in, Norwegian child and adolescent mental health outpatient clinics
*Specific research questions for the first aim are:*
  - Will children in families receiving MATCH exhibit lower levels of internalizing and externalizing problems at post-treatment compared to children in families who receive TAU, as measured by the Child Behavior Checklist (CBCL), Youth Self-report (YSR), and Child and Adolescent Trauma Screen (CATS)?
  - Will MATCH produce faster improvements in these domains than TAU and thus be more efficient (and cost-effective) than TAU, as measured by the Top Problem Assessment (TPA) and Behavior and Feelings Survey (BFS) across weekly assessments during treatment?
  - Will children in families receiving MATCH exhibit lower levels of internalizing and externalizing problems at follow-up (1 year after post-treatment assessment) compared to children in families receiving TAU, as measured by the CBCL, YSR, and CATS?
- To implement an empirically supported treatment (MATCH) in child and adolescent mental health outpatient clinics in Norway and to assess replicability of the findings from the research on MATCH in the US
- To evaluate whether potential treatment effects are associated with implementation quality and other moderators (e.g., age, gender, diagnosis, possible combinations of mental health problems)

## Methods/design

### Participants

Boys and girls aged 6–14.5 years (at intake) who have been referred to child and adolescent mental health outpatient clinics in Norway, because of symptoms of anxiety, depression, trauma and/or conduct problems, and their families.

#### Inclusion criteria

- Children and adolescents, 6 to 14.5 years of age at intake.

Scores above clinical cut-off scores on internalizing or externalizing scales of the CBCL/YSR or Child and Adolescent Trauma Screen (CATS)/ Assessment of Traumatic Experiences (Norwegian acronym, KATE) (see section on “Rationale for the use of cut-off scores” below)

- At least one of the parents has to be able to understand the consent form and answer the questionnaires and interviews in Norwegian. Both parents have to sign the consent forms in cases of joint custody

#### Exclusion criteria

- Children/youth with psychosis, intellectual disability, pervasive developmental disorder, anorexia, bulimia and/or who have been a perpetrator of sexual assault
- Children/youth who are acutely suicidal, or have carried out suicide attempts during the past 12 months. If a patient self-harms, the severity of that behavior is carefully evaluated on a case-by-case basis by the treating therapist together with the consultants. The case is excluded only if the self-harming is considered to be a threat to life or health. In these cases, the child or youth is referred to other treatments
- Children/youth who are to be treated solely for inattention and/or hyperactivity disorder (although ADHD comorbidity with any of the other conditions is not exclusionary)
- Youth (aged 13–14.5 years) who have shown repeated and serious antisocial/criminal behavior (e.g., severe threats to harm others, vandalism, burglary, arson, violence)
- Youth (aged 13–14.5 years) whose primary problem is substance abuse and for whom the substance abuse problems are expected to interfere with treatment. If the referral to the clinic does not mention any of the four problem areas, but substance abuse only, the family is not included in the study. Cases with substance abuse and one or more of the four problem areas are discussed case by case by the study team consisting of researchers and US and Norwegian clinicians
- Families can only have one child in the study at a time
- The child cannot receive active treatment simultaneously from another provider

### Measures

**Fig. 1:**
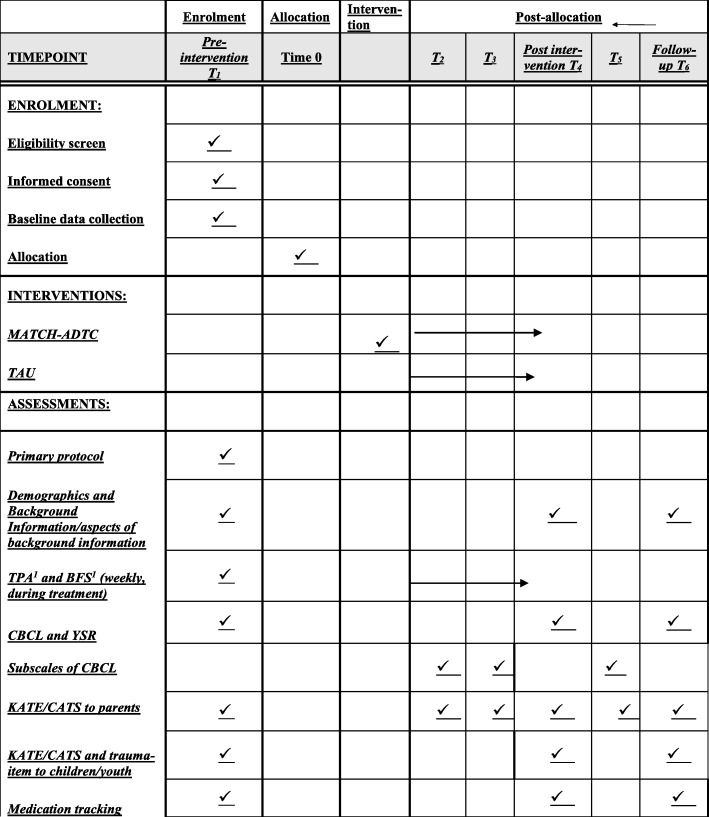
Schedule of enrollment, interventions, and assessments. ^1^*TPA* Top Problem Assessment, *BFS* Behavior and Feelings Survey, both part of the Progress Assessment in Therapy (PATH) system

Questionnaires and interviews are administered to the families in six waves, in addition to the weekly PATH questionnaire which is filled out by children (above the age of 8 years), parents and MATCH therapists between each session throughout the course of treatment. Therapists, supervisors, and team-leaders at each clinic also fill out questionnaires. See Fig. 1 and the description of measures for details. The pre-assessement (T1) is administered after receipt of consent and prior to randomization. At 3 and 6 months (T2 and T3, respectively) into treatment, a short assessment is administered to children and parents, and these are both time-invariant and thus occur at the same time for all families enrolled, regardless of group assignment or treatment progress. Shortly after treatment has concluded, families fill out the post-assessment (T4). The time at which the post-assessment is administered varies among families depending on how long the treatment period has lasted, and so this assessment is time-varying. At 15 months (T5) after treatment initiation, another short assessment is administered. The last assessment (T6), the follow-up, is administered 1 year after the end of treatment, and so is time-variant relating to T1 (because it depends on how long treatment lasted), but is time-invariant in relation to T4 (because it is carried out 1 year post treatment conclusion)

#### Demographics and Background Information

Families answer questions about age, gender, ethinicity, living arrangements, income, education, civil status and other children in the household. Background information also includes questions about service utilization, early childhood risk factors (e.g., prematurity) and how the child is doing in school. Demographics and background information is gathered at the pre-, post-, and follow-up assessments (T1,T4, and T6), though at the post- and follow-up assessments, only aspects that may change during the time of study participation are covered (e.g., marital status, service utilization, etc.)

#### Medication Tracking

We ask parents to indicate whether the child is, or has been, on any medication, which medication, and for how long. This measure is administered at pre-, post-, and follow-up (T1,T4, and T6).

#### Top Problem Assessment (TPA)

Children and parents fill out a severity rating (on a scale of 0 to 4) of the top three problems the youth and parent independently identify as most important to them in separate structured pre-treatment interviews. Psychometric analyses of the TPA have shown strong reliability, validity, and sensitivity to change during treatment in earlier studies of MATCH in the US [23, 24]. The TPA is part of the PATH system and ratings are collected weekly during treatment.

#### Behavior and Feelings Survey (BFS)

Children and parents fill out a 12-item measure of internalizing (six items; scores can range from 0 to 12), externalizing (six items; score range, 0–12), and total problems (12 items; score range, 0–24). This BFS was developed through psychometric testing of an item pool derived from the “top problems” identified by previous samples of clinically referred children and their parents. BFS scores show convergent and discriminant validity in relation to the appropriate scales of other child- and parent-report problem scales: the CBCL and YSR, the Strengths and Difficulties Questionnaire, and the Youth Outcome Questionnaire. The BFS is used with the PATH system, through which ratings can be collected weekly.

#### The Achenbach System of Empirically Based Assessment (ASEBA; CBCL and YSR)

The child’s behavior and symptomology are assessed by multi-informant ratings from caregivers and children on the CBCL [1] and YSR [1], respectively. This family of measures is one of the most widely used to assess child and adolescent emotional and behavioral functioning and has been validated in numerous studies (e.g., [12]). The instruments assess a broad array of behavioral and emotional manifestations of psychopathology, yielding eight syndrome scales of which the broad band scales “Internalizing” and “Externalizing” will be of particular importance in this study. CBCL and YSR are administered at the pre-, post-, and follow-up assessments. Parents also complete the internalizing and externalizing sub-scales of the CBCL at the 3-, 6-, and 15-month assessment points (waves 2, 3, and 5).

#### Assessment of Traumatic Experiences (Norwegian acronym, KATE) and Child and Adolescent Trauma Screen (CATS)

KATE is a 15-item index, asking children and parents to indicate, yes or no, whether the child has ever experienced any of the traumatic events listed. Items include natural disaster, terrorism, bullying, exposure to violence, sudden death of a loved one, physical and sexual abuse, etc. KATE was developed by the Norwegian Center for Violence and Traumatic Stress Studies [15]. If a child and/or a parent answers “yes” to any of the items on KATE, they will also be asked to fill out CATS, which measures the mental health consequences of trauma exposure [19]. The CATS is based on the *Diagnostic and Statistical Manual of Mental Disorders, 5th edition* (DSM-V) [2] criteria for post-traumatic stress disorder and targets responses such as re-experiencing, avoidance, and hyperarousal, dysphoria, and intrusion. Twenty items are rated on a 4-point scale ranging from “Never” to “Almost always.” At the end, five additional items capture psychosocial functioning by asking, yes or no, whether the previously rated mental health symptoms interfere with five key domains (getting along with others, school, hobbies, family, and overall happiness). KATE and, if indicated, CATS, are administered at all assessment points to parents. Children and youth complete KATE and CATS together with a therapist at the pre-assessment. At the post- and follow-up assessments, a single trauma item is administered, asking the child to indicate, yes or no, whether they have experienced a traumatic event since the start of the treatment. A short explanation of a traumatic event is provided. If “yes,” the child then completes the CATS.

#### KIDSCREEN-10

Health-related quality of life (HRQoL) is administered to children using the KIDSCREEN-10 [18], which is a short version of the KIDSCREEN-27 questionnaire. The recall period is 1 week and the items ask the child to rate among other things, their happiness, energy, fitness, and impression of parental fairness. Items are rated on a 5-point Likert scale ranging from “Not at all” to “Extremely.” The KIDSCREEN-10 is administered at the pre-, post-, and follow-up assessments.

#### Hopkins Symptom Checklist (HSCL-5)

Hopkins Symptom Check List-5 (HSCL-5, [20]) measures parental mental health, with five questions targeting anxious and depressive symptoms. The Checklist has a 1-week recall period with response alternatives ranging from 1 to 4, in which higher scores indicate greater symptomology. The HSCL-5 is administered to parents at the pre-, post-, and follow-up assessments.

#### Working Alliance Inventory (WAI)

Therapeutic alliance is assessed by the 12-item Short Form of the Working Alliance Inventory [21]. Responses are collected from parents who are asked to rate each statement (e.g., “I trust the therapist’s ability to help me”) on a 7-point Likert scale ranging from 1 (never) to 7 (always). Higher scores indicate greater therapeutic alliance. WAI is administered at the 3-month, 6-month, and post-assessments.

#### Family Satisfaction Survey

Parents and therapists complete the Family Satisfaction Survey (Lubrecht J: Family Satisfaction Survey, unpublished), a 12-item questionnaire, at treatment termination. Caregivers are asked to rate questions relating to treatment effectiveness and whether they would recommend the treatment to others, on a 4-point Likert scale. Higher scores indicate greater treatment satisfaction. Therapists complete the therapist version of the survey asking similar questions. The Norwegian-translated versions have been used in an earlier clinical trial with Norwegian youth and their families [16].

#### Experience with Evidence-Based Practice (E-EBP)

Clinicians are asked to indicate the extent to which their previous clinical work has included evidence-based practices (EBP) or programs. Questions include which type of EBP number of patients treated in EBT, number of years working with one or more EBTs and the degree of training and guidance received. The E-EBP is administered to clinicians at the pre-assessment.

#### End-of treatment Questionnaire

Clinicians are asked, at the completion of each individual treatment, questions regarding whether there were any barriers to the family’s participation in treatment, support from other services, and dosage.

#### Implementation quality

Implementation quality at the organizational level is assessed by conducting interviews with clinic supervisors, team leaders and therapists in the participating clinics. The interview for clinic supervisors and team leaders consists of 27 items targeting uptake, satisfaction, challenges with and integration of MATCH in their clinics, and whether any organizational adjustments had to be made to comply with MATCH. Therapists are asked to respond to questions regarding satisfaction with training and ongoing consultation, leadership support, and organizational adjustments. Clinic supervisors and team leaders are interviewed three times during the study period, twice mid-study and once at the end of the study. Therapists are interviewed twice, once mid-study and once at the end of the study. The interview for both leaders and therapists is based on the Fixsen et al. [9] synthesis on implementation research.

#### Quality of treatment delivery and fidelity

All therapy sessions are videotaped. The focus of the recordings is the therapists’ delivery of treatment. Both MATCH and TAU therapists videotape their sessions. Data from these videotaped sessions will be used to assess the degree to which MATCH is delivered with competent adherence (for the MATCH therapists) and how well therapists in both conditions are able to involve the families, communicate with families, and establish a treatment environment conducive to therapeutic gains. All use of the videotaped material will entail coding of the films. Coding of videotapes are, however, contingent upon funding.

### Design and power

The trial is a randomized controlled effectiveness trial conducted in existing child and adolescent outpatient clinics in Norway. Participants are randomized within each site, following a computer-generated list with each participant having a 50% chance of being assigned to either group. The results of the randomization are provided to the clinic for each new case. The process is as follows:

- *Referral*: children and their families are referred to the participating clinics (primarily by either their general physician, head of the municipal child welfare agency, or community psychologist) for child symptoms of anxiety, depression, trauma-related problems and/or conduct problems. Children and families are assessed for eligibility for the study based on these referral reasons, and on whether they have significant symptom levels meeting study cut-offs at the time of screening (see item c and “Rationale for the use of cut-off scores” below)
- *Consent*: once found eligible at this stage, families are informed of the trial by the contact person at the clinic. If the family consents, their contact information is given to the Norwegian Center for Child Behavioral Development (NCCBD) which then registers the information and contacts the family to provide more information about the trial and to ask whether the family wants to participate. The family is informed briefly about the treatment options and that random assignment is involved. If the family agrees, a consent letter, along with a brief study description (including contact information of the study personnel), is sent to the family. The NCCBD then registers receipt of a signed consent from the family. A phone call is made to the family within a few days to make sure that they have received the letter in the mail. If the NCCBD has not received a signed consent within 2 weeks, another phone call is made to the family, to allow for questions and to urge interested families to sign and send in the consent form.
- *Pre-assessment and decisions about inclusion to the trial*: upon receipt of a signed consent form from the family, the pre-intervention assessment (wave 1) is sent out to the family This is done approximately 2 weeks prior to the date set for their first consultation at the clinic. A reminder is sent out to the family after 1 week, and is followed up by weekly phone calls if they have not submitted the completed assessment. The pre-intervention assessment, on which scores are based to assess eligibility to the trial, include the CBCL, YSR, KATE and if relevant (given the scores on the KATE), the child and parent version of CATS. Children between the ages of 8 and 11 years complete these questionnaires at the clinic together with a clinician. The family is eligible if the child’s scores are at or above clinical cut-off scores.
- *Further assessment by the clinics and setting the primary protocol*: the clinics receive the summary scores on the CBCL, YSR, KATE, and CATS from the NCCBD. In some cases, the clinics administer these questionnaires directly to the families as part of their routine, and in these cases, the NCCBD receives the results from the clinic(s), after collected consent. For children with T-scores above the cut-off, a therapist at the clinic conducts a TPA. Based on the result from these assessments, MATCH consultants make the decision about the child’s primary protocol to be tried first (primary problem area). MATCH consists of four treatment protocols, one for each problem area; anxiety, depression, trauma, and conduct problems
- *Randomization*: once inclusion to the study and primary protocol are determined, the family is randomized to either the MATCH condition or the TAU condition. Information about the outcome of the randomization procedure is then provided to the clinic. The outcome of the randomization then triggers two events: (1) the dates for the time-invariant assessment points (i.e., the 3-, 6-, and 15-month assessments) and (2) the clinics being alerted to inform the NCCBD of who the therapist for the family will be, along with their contact information
- *Registration in PATH*: families are then added to the PATH system a week prior to their first meeting with their appointed therapist. The families receive an email on the Sunday of each week asking the parent and the child (if they are over 8 years old) to fill out a questionnaire, consisting of the TPA and the BFS. The TPA and the BFS are collected weekly (or between each session) as part of the PATH system of user feedback

#### Rationale for the use of cut-off scores in assessing eligibility for study participants

Potential study participants are first assessed with the Achenbach System of Empirically Based Assessment (ASEBA:CBCL and YSR) and CATS. Participants who score at or above 1 standard deviation on at least one of the broadband scales (externalizing and internalizing, a T-score at or above 60) of either the CBCL or the YSR, are defined as meeting the inclusion criteria. Additionally, any patient scoring at or above 1.5 standard deviations on any of the relevant sub-scales of the CBCL or the YSR (e.g., aggressive behaviors or anxious-depressed) are accepted for study participation (a T-score at or above 65).

Moreover, all patients having a score at or above 15 on CATS are also deemed eligible for study participation. This cut-off is based on recommendations from the Norwegian translators and expert users of CATS (Norwegian Center for Violence and Traumatic Stress Studies, NKVTS). CATS is a revision of the Child PTSD Symptom Scale (CPSS), with some added items to cover the diagnostic criteria of DSM-V. A clinical cut-off for CPSS is a score of 11 or above [10], but because the CATS contains four additional items to be scored from 0 to 4, the cut-off for CATS is set to 15.

#### Primary outcomes

- CBCL/YSR
- CATS
- Weekly PATH scores: TPA and BFS (throughout treatment)
- Time in therapy

#### Secondary outcomes

- KIDSCREEN-10
- SCL-5
- Treatment satisfaction
- Quality of treatment delivery and fidelity

#### All additional measures administered during the trial

- Demographics and background information
- Child medication
- Therapist alliance
- Experience with evidence-based practice
- Implementation quality

### Procedures

#### Intervention/Route of administration

When the child and family have been randomized to a MATCH therapist, the therapist will also be informed regarding the child’s primary protocol. The therapist will bring the case to the consultation group led by a US consultant and receive consultation on how to start the treatment. The therapist will arrange with the family when to meet and the treatment will then be delivered at the clinic. The assigned primary protocol (either anxiety, trauma, depression, and/or conduct) will lead the sequence of modules used. A treatment will always involve more than one module. To manage clinical interference that requires deviation from the primary protocol, the therapist, in agreement with the consultant, can choose among several of the 35 modules. If there are any remaining difficulties that need to be addressed after the primary protocol has been completed, the therapist can start a new protocol.

Children and families in the comparison group, who receive TAU, are offered the treatment normally delivered in the clinic. The content and structure of TAU vary among clinics and among individual therapists. TAU therapists may deliver family-based therapy based on an eclectic approach; they may offer a standard, single-syndrome EBT (for example, Coping Cat or Parent Management Training) or enroll the child or family in group therapy. Families in both conditions complete PATH, but only MATCH therapists will have access to the families’ responses. Therapy sessions of both conditions are videotaped, with the exception of children and families in the TAU condition who take part in group-based therapy (due to data confidentiality restrictions on children and families who are not part of the study). See the trial Standard Protocol Items: Recommendations for Interventional Trials (SPIRIT) Checklist for details (in Additional file 1).

#### Interventions

MATCH-ADTC is the experimental condition, as described above. Children and families in the TAU condition will receive treatment as it is usually delivered in the clinic.

#### Treatment permitted and not permitted during the trial

Children and families in the MATCH condition will receive the MATCH treatment only. The sole exception is that the children may receive a medication regimen deemed necessary by the clinician. Medication use is registered and tracked throughout the treatment period for all participants. No restrictions are imposed on what is offered to the children and families in the TAU condition. There are also no restrictions for participants in either condition regarding prior treatment or prior participation in any EBTs.

#### Procedures for monitoring participant compliance

Children and parents answer the weekly PATH questionnaire, which assesses treatment progress. Questionnaires of the PATH system are sent out automatically every Sunday, and a follow-up email is sent every Tuesday. If families fail to fill out questions in the PATH system, they are reminded to do so by members of the research team. The research team at the NCCBD also remind children and families to fill out the questionnaires at the various data collection waves. This is done regularly by phone, text message, or email.

#### Dosage

The MATCH treatment is delivered in weekly or bi-weekly sessions. Each session lasts 45–60 min. Although parents are usually more involved when conduct problems are the primary treatment focus, parent involvement is also emphasized when the treatment targets anxiety, depression, or trauma. There are separate modules for psychoeducation for parents in all the treatment protocols. In addition, when children meet with their therapists alone, parents are invited to participate in the last 10–15 min of the sessions to make sure they get a proper understanding of the skill the child is about to learn, and of how to support the child in doing the homework assignment for the coming week. It is a pronounced goal that the parents eventually take over functions that the therapist has had, in terms of facilitating and motivating the child to use new skills and to continue practicing.

#### Treatment period

There is not a set number of sessions; the MATCH therapist follows the flowchart for the primary protocol. The therapist moves on to a new module when the content of the module in question has been properly covered or when the clinical concern addressed by the module has resolved. If there is interference that prohibits the therapist from following the prescribed sequence, the therapist can use modules from a different protocol until the interference is resolved. Treatment will end when the PATH data show reasonable progress, combined with the clinical judgment that most of the improvement that is likely to happen, has been achieved. As in all treatments, some cases will not have the expected progress, and the therapist will discuss with the consultant whether another approach would be appropriate. In an article reporting on the effects of MATCH in the US, the average number of therapy sessions was 30.02 and because most sessions are conducted weekly, the average time period for treatment was about 7.5 months [23]. There are no accurate statistics available indicating what the average time in therapy is for TAU in Norway. One of the goals of this study is, however, to compare time in treatment between the two conditions.

#### Description of stopping rules (discontinuation criteria)

##### Individuals

If the therapist (in agreement with the clinic supervisor) uncovers acute suicidality, psychosis, self-harm, abuse, or maltreatment in the family, any of the exclusion criteria, or other situations that render the MATCH treatment or regular practice not viable or safe. If the child is placed out of home and/or parents’ lose custody of the child. Withdrawn consent from the study or treatment.

##### Parts of the trial

If a site or a therapist does not follow through on trial requirements or agreements.

##### The entire trial

Withdrawn government funding (to the NCCBD), breach of ethical standards or regulations. If any of the participating mental health outpatient clinics uncovers any serious adverse events due to the treatment, leading them to conclude that it is not safe to offer MATCH to patients.

### Statistical analyses

We plan to conduct several analyses, each of which will answer different hypotheses and have its own type of statistical analysis.

#### For main effects

The time-variant assessments, that is, the pre-, post-, and follow-up assessments, will be analyzed using descriptive statistics and general linear models (GLM), controlling for age, gender, type of problem area, and family background information (e.g., SES).

#### Timing of analyses

We will analyze treatment effect when the last family has completed the post-assessment battery. We will start analyzing the follow-up data when the last family has completed the follow-up assessment.

#### For effects according to time-invariant assessments

For analyses of trajectories of change, we will employ mixed-effects regression analyses and growth curve analyses (for the pre, 3-, 6-, and 15-month data and PATH data).

#### For subgroup analyses

Descriptive statistics, general linear models (GLM), and multiple group analyses.

#### For associations of implementation quality and fidelity

Correlation, regression, and interactional models.

#### For predictors of treatment outcomes (other than treatment condition**)**

Structural equation models, regression, path modeling.

#### Level of significance to be used

In most analyses, a significance level of α = .05 will be used for main effects analyses, mediation models, growth curve models, and general models of association. We will use more stringent alpha levels in subsequent analyses in cases where many variables are entered into prediction models, to reduce the risk of Type 1 error.

### Sample size

#### The number of subjects planned to be enrolled

We plan to recruit 280 children and their families. Based on a power analysis for the analysis of main effect, we concluded that a sample size of 280 will detect an effect size of 0.3, provided power of .80, at an alpha level of .05.

#### The number of participants projected at each site

The number of families to be recruited at each site was calculated based on the number of therapists trained at each site, with the expectation that each MATCH therapist would contribute with at least six cases each to the study, matched by an equal number of cases from TAU therapists. Six clinics participate at the present. Originally, one more outpatient clinic was recruited to take part in this trial. In the fall of 2016, however, it became clear that this clinic was not able to fulfill the requirements of participation and was, therefore, excluded from the trial, from therapist consultation, from the use of the PATH system, and all other MATCH elements. This decision was reached in agreement with the management at the clinic in question. We had originally planned to recruit 36 families from this site. Thus, the remaining six clinics have been asked to recruit, between them, the remaining families to compensate for the loss. As of fall 2017, we are in the process of recruiting two or three more clinics.

#### The selection of participants to be included in the analyses

We will answer two important research questions: First, to answer the question of “Does MATCH work?” we will employ a per-protocol design; thus only those children and families who have gone through treatment will be included. Second, the question of whether “MATCH makes a difference in existing clinics” is best approached via an intention-to-treat design (ITT), in which all families randomized will be included in the analyses, regardless of whether they entered or completed treatment. Thus, we will analyze data using both ITT and per-protocol designs in an effort to answer these two overall questions.

### Monitoring and safety

#### Procedures for recording of adverse events

The clinics follow internal procedures for detecting adverse events. The clinics continuously assess the proper and secure treatment of patients and their families, as they would normally do. The clinics will inform the NCCBD if any adverse events occur.

#### The type and duration of the follow-up of participants after adverse events

The clinics follow internal procedures for following up families in the case of adverse events.

#### Data Monitoring Committee (DMC)

This study is an effectiveness trial, conducted in existing child and youth mental health clinics, not in a research facility. The intervention is psychotherapeutic and is considered low risk and no more intrusive than what is being delivered to children and families in the clinics as part of their regular treatment delivery. The participating clinics have internal procedures for detecting, reporting, and following up on any adverse events in their patients. The study does not pose any restrictions on the clinics’ internal procedures for handling adverse events or from offering another treatment if deemed appropriate. In the event of the clinic terminating a MATCH treatment, the research team at the NCCBD will be informed, and post-assessment will be conducted. The trial is not blinded, as the therapists know what kind of treatment they offer the children and their families. MATCH therapists are routinely guided by US consultants with follow-up assistance from Norwegian co-consultants throughout the treatment. Interim analyses will not be conducted, in order not to bias the progression of the study.

Carandang et al. [5] reported on a set of issues to consider for mental health researchers when deciding whether to appoint a DMC for a mental health study. These issues included (1) risk of the psychiatric disorders (e.g., suicidal risk), (2) features of treatment (whether it is pharmacological), (3) research environment (extend of experience), (4) lack of information on the study population (e.g., comorbidity), and (5) lack of information about the treatment (e.g., novel and without prior results). The study population in the current study can certainly be considered vulnerable; however, information about suicide attempts (and suicidal ideation) and psychosis are collected and excluded from participation, the intervention is psychotherapeutic and is considered low risk, and the research environment consists of experienced researchers who have conducted several randomized trials with similar populations of children and youth. The researchers and the treating therapist(s) know a great deal about the children and their family. The intervention consists of known and well-tested treatment components, and it has been implemented in other settings with promising results. For these reasons, the current study has not appointed a DMC.

### Access to source data

The NCCBD (the sponsor) ensures that the investigator/institution will permit trial-related monitoring, audits, reviews, and regulatory inspections, by providing direct access to source data/documents if needed, and that such inspections do not violate the rights and/or anonymity of trial participants, including children, their families or therapists or other clinic employees.

The Regional Committees for Medical and Health Research Ethics require that all research data and participant contact information are stored for 5 years after project termination in order to make possible audit by the Norwegian Board of Health Supervision.

## Discussion

### Guidance and Monitoring of MATCH treatment

Monitoring of treatment progress in therapy is done via the PATH system, where the therapists fill out a session note for every session: Session number, date, person(s) present, MATCH modules used, additional information (e.g., no modules used, crisis of the week), session activities (prior homework completed, new homework assigned, role play, in vivo exercises, summary of session, plan for next session).

MATCH therapists underwent an initial 6-day intensive training program led by US MATCH consultants, focusing on rationale for, and basic principles in, MATCH, and the nature, prevalence, theoretical underpinnings, and treatment of the various conditions targeted by the treatment model (anxiety, depression, trauma, and conduct problems). All modules were presented in-depth, and supported by modeling and/or video demonstrations from real cases, and role-playing. Further development of the therapists’ competency is in the format of on-the-job training, with weekly “prep calls” with a Norwegian co-consultant, and weekly group consultation with US consultants. The training by the US consultants was led in English.

### Quality assurance

Quality assurance of MATCH therapists is achieved by weekly online video meetings with the US consultants and the Norwegian co-consultants. During the consultation, which is based both on information from the PATH data and on a short summary of what happened in the last session provided by the therapist, the consultant asks specific queries to monitor whether the therapist adheres to the treatment principles of MATCH. The focus of the consultation is to plan treatment, based both on the families’ feedback in PATH and the therapists’ clinical judgments, in accordance with the flowchart for the primary protocol. Obstacles and problem-solving strategies are discussed, and the therapist and US consultant arrive at a plan for the next session. The consultant will often model new procedures in these Skype meetings. In addition, there is a weekly preparation meeting with the Norwegian co-consultant in which PATH entries are reviewed, and the therapists and their designated co-consultant role-play in Norwegian to prepare the delivery of new modules and to ensure adherence to the model. The consultation by US consultants is led in English and the pre-consultation call is led in Norwegian by the Norwegian consultants. The consultation also addresses basic principles and theoretical understanding behind the interventions, and individual tailoring of the treatment, including age-appropriate adaptation.

The format of the consultation is in-group case supervision guidance. Consultation groups consist of two to four therapists, together with a US consultant and a Norwegian co-consultant. Case-relevant modules are reviewed and often modeled by the US consultant. The consultation also addresses basic principles and theoretical understanding behind the interventions, and individual tailoring of the treatment, including age-appropriate adaptation.

The Norwegian co-consultant sets up role-plays for the therapists in the following prep call, in order for the therapists to train in new skills, and for the co-consultant to give feedback regarding performance.

### Ethics

Before the start of the research project, the trial was reviewed and approved by the Regional Committees for Medical and Health Research Ethics, Southern and Eastern Norway (REK South East). There are ethical concerns related to offering families an intervention that has not yet been evaluated in a Norwegian context. The MATCH treatment procedure has shown promising results in the US and its treatment principles and procedures are well aligned with treatment that the clinics would like to deliver to children and families in their care. The random assignment of this trial elicits ethical considerations as some families may be disappointed by their assignment to the TAU group. These families are, as are all the families participating in the study, assured that they will receive treatment within the child and adolescent mental health clinics, and these clinics will follow regular safety procedures and guidelines for treatment. The goal in both treatment conditions is to ensure that the participants feel cared for and respected, and that treatment will help the child and family in question. Completing the questionnaires takes some time, and it may incur an additional burden to answer sensitive questions. Young respondents are assisted in filling out the questionnaires, if needed. We are limiting the questionnaires to include assessments that are deemed important in terms of evaluating the effectiveness of the intervention, only. Participants receive a moderate financial compensation for the time they use to complete questionnaires. This payment is also meant to promote participant retention, and the amount increases with the number of data collection waves.

The procedures of the data collection, and the handling and storage of data fulfill security, anonymity and confidentiality requirements. Confidentiality and anonymity are secured in any publication from this trial, popular or scientific. By conducting this study, we intend to learn more about how to offer effective treatment to vulnerable children and youth.

### Facilitative administrative supports

Leaders of the participating clinics have been involved in this project through meetings with the implementation and research staff of the NCCBD throughout the planning and recruitment phase, as well as for troubleshooting and local adaptations in the initial implementation phase. The administration of the participating clinics have made it possible for their therapists to set aside time for training and consultations. In cases of modifications to the protocol, clinics and participating therapists at the clinics will be called to a meeting discussing how to best handle such changes.

The NCCBD has been in contact with all levels of the administration, and both clinic leaders and team leaders are informed that some of their therapists participate in the project. The therapists have the possibility of discussing their MATCH cases with their team leader in weekly team meetings, but the team leaders are not themselves trained in MATCH and most of them know less about the intervention.

The results from this trial will produce knowledge about both how MATCH treatment is delivered in existing child and adolescent outpatient clinics in Norway, and the content and delivery of TAU. Most importantly, the results from this study may lead to better treatment of complex problems in children, and to the development of knowledge that can be used by clinicians who work with children and families in Norway.

## Trial status

This is protocol version number 2. Protocol version 1 is written in Norwegian only.

Recruitment began in March 2016 and will go on until the beginning of 2019.

## Additional file

Additional file 1: Standard Protocol Items: Recommendations for Interventional Trials (SPIRIT) 2013 Checklist: Evaluating Modular Approach to Therapy for CHildren with Anxiety, Depression, Trauma and Conduct problems (MATCH-ADTC) in Norwegian child and adolescent outpatient clinics: study protocol for a randomized controlled trial. (DOC 122 kb)

## Abbreviations

BFS: Behavior and Feelings Survey
EBP: Evidence-based practice
EBT: Evidence-based treatment
MATCH-ADTC: Modular Approach to Treatment for CHildren with Anxiety, Depression, Trauma and Conduct problems
TAU: Treatment as usual
TPA: Top Problem Assessment
